# Oxygen Vacancies in Oxide Nanoclusters: When Silica Is More Reducible Than Titania

**DOI:** 10.3389/fchem.2019.00037

**Published:** 2019-02-07

**Authors:** Andi Cuko, Stefan T. Bromley, Monica Calatayud

**Affiliations:** ^1^Departament de Ciència de Materials i Química Física, Institut de Química Teòrica i Computacional (IQTCUB), Universitat de Barcelona, Barcelona, Spain; ^2^CNRS, Laboratoire de Chimie Théorique, LCT, Sorbonne Université, Paris, France; ^3^Institució Catalana de Recerca i Estudis Avançats (ICREA), Barcelona, Spain

**Keywords:** TiO_2_, SiO_2_, titanosilicates, nanoclusters, oxygen vacancy, reducibility

## Abstract

Oxygen vacancies are related to specific optical, conductivity and magnetic properties in macroscopic SiO_2_ and TiO_2_ compounds. As such, the ease with which oxygen vacancies form often determines the application potential of these materials in many technological fields. However, little is known about the role of oxygen vacancies in nanosized materials. In this work we compute the energies to create oxygen vacancies in highly stable nanoclusters of (TiO_2_)_N_, (SiO_2_)_N_, and mixed (Ti_x_Si_1−x_O_2_)_N_ for sizes between *N* = 2 and *N* = 24 units. Contrary to the results for bulk and surfaces, we predict that removing an oxygen atom from global minima silica clusters is energetically more favorable than from the respective titania species. This unexpected chemical behavior is clearly linked to the inherent presence of terminal unsaturated oxygens at these nanoscale systems. In order to fully characterize our findings, we provide an extensive set of descriptors (oxygen vacancy formation energy, electron localization, density of states, relaxation energy, and geometry) that can be used to compare our results with those for other compositions and sizes. Our results will help in the search of novel nanomaterials for technological and scientific applications such as heterogeneous catalysis, electronics, and cluster chemistry.

## Introduction

Metal oxides are an important class of materials in many scientific and technological fields. Silica and titania are abundant compounds and play crucial roles in geology (Howie, 1964; Lakshtanov et al., 2007; Lyle et al., 2015), astrochemistry (Lambert et al., 2012; Lee et al., 2015; Komatsu et al., 2018), environmental and chemical sciences (Joo et al., 2014; Kapilashrami et al., 2014), biology and medicine (Mal et al., 2003; Nguyen et al., 2005; Das et al., 2009; Bawazer et al., 2012; Lucky et al., 2016; Saroj and Rajput, 2018). Their ability to exchange oxygen drastically determines their properties. In the case of TiO_2_, the loss of lattice oxygen results in a change of color from white to blue, the appearance of magnetism, and is connected with photocatalytic properties (Houlihan et al., 1975; Diebold, 2003; Di Valentin et al., 2006; Na-Phattalung et al., 2006; Vásquez et al., 2016; Li et al., 2018). On the other hand, oxygen vacancies present in amorphous silica have been reported to modify its electronic and optical properties (Rudra and Fowler, 1987; Skuja, 1998; Manveer et al., 2017). Oxygen substoichiometry in silica-based materials is linked to dielectric breakdown and electroforming in resistive random access memory devices (McPherson and Mogul, 1998; Yao et al., 2010; Wang et al., 2014; Mehonic et al., 2018). Whereas, the effect of creating an oxygen vacancy in bulk and surfaces of both titania and silica is well-reported, little is known about the role these defects may play in nanosized materials and clusters. By means of density functional theory (DFT) based calculations, we characterise the energetics of creating an oxygen vacancy in silica, titania and titanosilicate clusters recently derived from global optimization studies. Low energy clusters can be generated with cluster beam experiments and stabilized by soft landing techniques. Such studies are increasingly looking into the reactivity of the cluster species formed for technologically important reactions (Yin and Bernstein, 2014; Vajda and White, 2015; Sun et al., 2016). With our work we hope to elucidate the reactivity of such nanoclusters as estimated through their reduction by oxygen removal.

From a computational point of view, the energetic cost of removing a neutral oxygen atom from a stoichiometric metal oxide can be calculated with the following equation:

$$\begin{array}{rcl} {\text{M}}_{\text{n}}{\text{O}}_{2\text{n}}\; & \to & \;{\text{M}}_{\text{n}}{\text{O}}_{2\text{n}-1}+1/2\;{\text{O}}_{2} \end{array}$$

(1) $$\begin{array}{rcl} {\text{E}}_{\text{vac}} & = & \text{E}{\text{M}}_{\text{n}}{\text{O}}_{2\text{n}-1}+1/2\text{E}{\text{O}}_{2}-\text{E}{\text{M}}_{\text{n}}{\text{O}}_{2\text{n}} \end{array}$$

A reducible oxide like titania tends to have small (positive) E_vac_ values, while the energy to form an oxygen vacancy in an irreducible oxide like silica is relatively higher. E_vac_ values can thus be used to compare different systems and provide an initial ranking of reducibility (Ganduglia-Pirovano et al., 2007; Deml et al., 2015; Helali et al., 2017). Typical values for bulk and surface *E*_vac_ values in rutile and anatase TiO_2_ are 3.7–4.5 eV whereas for SiO_2_ the values are much higher, 5–8 eV (see Sushko et al., 2005). In addition, the localization of the electrons remaining after the removal of the oxygen in the lattice provides crucial information related to the electronic structure of the defective material (Helali et al., 2017):

(2) $$\begin{array}{r} M^{\text{n+}}-O^{2-}-M^{\text{n+}}\to M^{\text{n+}}-2e^{-}-M^{\text{n+}}+\frac{1}{2}O_{2} \end{array}$$

It is commonly accepted that removing an oxygen atom from TiO_2_ leads to the formation of paramagnetic Ti^3+^ sites in both bulk and surfaces (Houlihan et al., 1975; Di Valentin et al., 2006; Li et al., 2018). The electronic state of reduced titania systems is typically open-shell. In amorphous SiO_2_ and quartz the removal of a lattice oxygen results in the formation of a Si-Si covalent bond where the electron pair is stabilized by two Si sites (Sushko et al., 2005). In the quartz surface the removal of an oxygen atom leads to an electron pair localized on a surface silicon site (Silvi and D'Arco, 2006; Causà et al., 2015). This typically leads to electronic states that are closed-shell.

We focus on the effects that the system size reduction to the nanoscale has on the type of oxygen defects exhibited and their resultant E_vac_ values. As has been previously reported for ZrO_2_ (Ruiz Puigdollers et al., 2016a,b), CeO_2_ (Kozlov et al., 2015), TiO_2_ (Morales-García et al., 2018), this may result in a significant change in the materials' properties. Specifically, in this work we investigate the energetic, structural and electronic properties of silica, titania and mixed titanosilicates as regards their tendency to lose neutral oxygen atoms. These properties are then compared between the different oxides and with respect to the system size, including up to the bulk. At the nanoscale level, we consider a set of globally optimized silica and titania clusters with sizes between 2 and 24 MO_2_ units. Moreover, we compare our results for these pure oxides with a comparative set of titanosilicate clusters for the size of 10 MO_2_ units. At the bulk level, we consider alpha quartz and rutile phases, respectively, for silica and titania, which are the two most stable phases under ambient conditions. Such systems are then reduced by a single and neutral oxygen vacancy and the results are analyzed to elucidate the role of cluster size, structure and oxide type with respect to the formation of oxygen vacancies.

## Methodology

All nanocluster structures selected in this study correspond to the lowest energy global minima candidates currently reported in the literature as derived by us and/or other authors: SiO_2_ systems are derived from Flikkema and Bromley (2003, 2004), Lu et al. (2003), Bromley and Flikkema (2005), TiO_2_ systems from Hamad et al. (2005), Woodley et al. (2006), Calatayud et al. (2008a), Syzgantseva et al. (2011), Chen and Dixon (2013), Bhattacharya et al. (2015), Aguilera-Granja et al. (2016), Lamiel-Garcia et al. (2017), and finally mixed titanosilicates are taken from Cuko et al. (2018). A two-step procedure is followed to compute E_vac_ (Equation 1): (i) a selected oxygen atom is removed from a structurally frozen stoichiometric nanocluster and the pre-removal/post-removal energy difference is defined as E_unrel_, (ii) the post-removal non-stoichiometric nanocluster structure is then fully optimized, with the pre-optimized/post-optimized energy difference defined as E_vac_. The relaxation energy E_rel_ is then derived as the difference between E_unrel_ and E_vac_. For the smaller sizes, *N* = 2–10, every symmetrically distinct oxygen site was tested and only the most representative ones were reported. For larger sizes, an exhaustive approach was found to be too computationally demanding and a sampling of approximately 10 oxygen sites was performed, always including all symmetrically non-equivalent terminating oxygens. All DFT based calculations involving reduced nanoclusters were carried out using a spin-polarized formalism, employing the PBE0 functional (Adamo and Barone, 1999) and a tier-1/tight numerical basis set as implemented in the FHI-AIMS code (Blum et al., 2009). In most of the calculations, we did not force the spin multiplicity of the system. However, in a few cases, especially for TiO_2_ systems, in order to overcome convergence problems, we forced the spin multiplicity to be either an open shell singlet or a triplet. Recently, room temperature ferromagnetism has been reported for zirconia nanoparticles (Rahman et al., 2016; Albanese et al., 2018). This effect might also take place in titania and could be studied by considering multiple vacancies in future works.

## Results and Discussion

### Pure (SiO_2_)_N_ Nanoclusters

**Figure 1:**
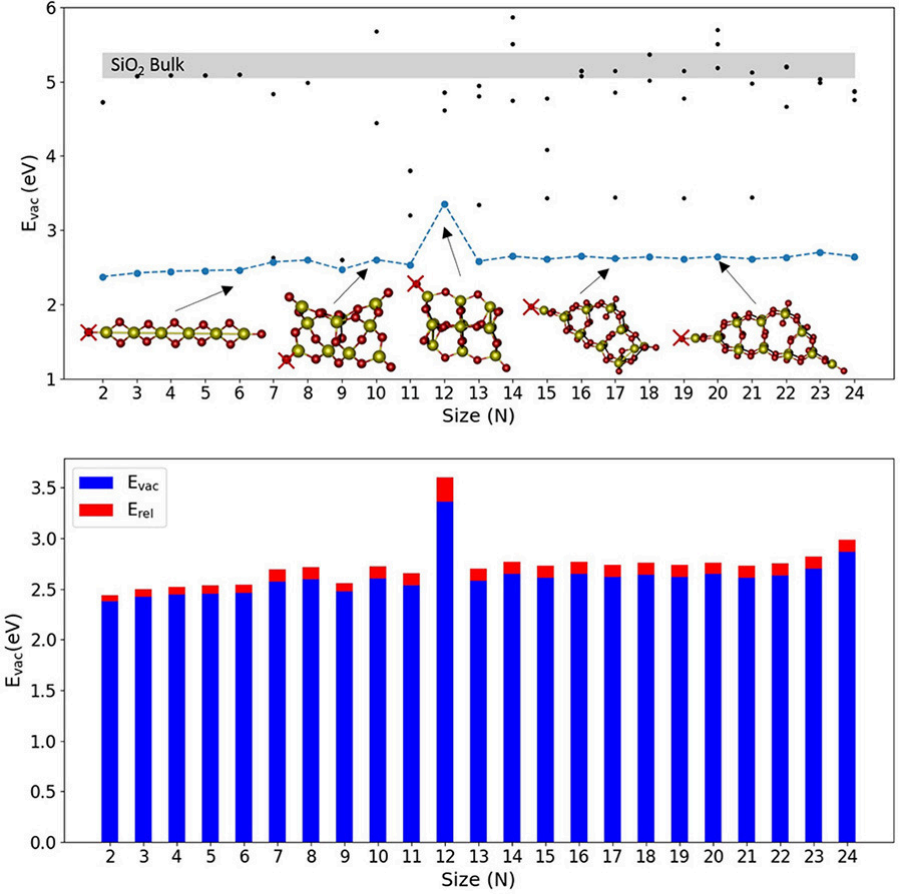
Top: E_vac_ vs. N plot of global minimum isomers for selected (SiO_2_)_N_ nanocluster sizes. Blue line is relative to lowest E_vac_ obtained associated with the removal of O atoms indicated with red crosses. Black points indicate a selection of E_vac_ values obtained from the removal of different sites of O atoms. Light gray region indicates the range of E_vac_ values calculated for α-quartz SiO_2_ bulk. Bottom, the corresponding oxygen vacancy formation energy values, E_vac_ (blue), and relaxation energy values, E_rel_ (red), with respect to nanocluster size.

Figure 1 shows the calculated values of E_vac_ for the lowest energy SiO_2_ nanoclusters together with some selected nanocluster structures. Figure S1 shows the structures of all stoichiometric silica nanoclusters used in this work and Table S1 includes the corresponding values of E_vac_, E_unrel_, and E_rel_ for the most stable reduced systems. Generally, stoichiometric silica global minimum nanoclusters tend to possess quite open symmetric structures and typically have between 2 and 4 unsaturated (terminal) oxygen sites. The majority of these structures present silanone centers, which are triply-coordinated planar Si centers involving a terminal oxygen atom (i.e., formally >Si=O, but arguably more accurately >Si^+^-O^−^) (Avakyan et al., 2006; Zwijnenburg et al., 2009). For nearly all the silica nanoclusters considered, the terminal oxygen atoms at these sites are found to cost the least energy to be removed, 2.6–2.8 eV, with E_vac_ increasing slightly and monotonically with the size. The (SiO_2_)_12_ nanocluster is the only exception as it only displays non-bridging oxygen (NBO) terminal species (i.e., ≡Si–O species where Si is 4-fold coordinated) rather than silanones. Removing the terminal oxygen atom of an NBO center results in a higher E_vac_ value (3.60 eV) as compared with silanone defects (2.6–2.8 eV). We note that the values of E_vac_ for the removal of oxygen atoms at both silanone and NBO defects are much lower than those for the removal of 2-fold coordinated oxygen atoms in the corresponding bulk systems. From our calculations, for example, we obtained E_vac_ values between 5.06–5.39 eV for a-quartz with a range of vacancy concentrations between 1/24 and 1/3 vacancies per SiO_2_ units. These values are also consistent with other work where an E_vac_ value of 5.15 eV was obtained using plane-wave based DFT calculations at the PBE level for the alpha-quartz structure with a vacancy concentration of 1/48 (Helali et al., 2017). We note that, removing a 2-fold oxygen atom in our nanoclusters typically costs between 4.2 and 5.7 eV (see Figure S2 which shows selected values for Si_10_O_20_), which is comparable to the E_vac_ values obtained for bulk alpha quartz. This confirms that the terminal silanone oxygens are least energetically costly in silica nanoclusters. From a structural point of view, reducing a silanone defective site in a nanocluster leads to a 2-fold Si site displaying a Si-O distance of 1.71 Å. This is slightly longer than the Si-O bond length of 1.62 Å as calculated by Pacchioni and Ferrario (1998) for a 2-fold coordinated Si defect in alpha quartz using a cluster approach at both wave function theory level employing unrestricted Hartree Fock, and DFT level employing the hybrid B3LYP functional. Further comparing our results with these bulk cluster calculations, in our clusters the O-Si-O angle of a reduced silanone site is found to be between 87 and 111°, depending on whether the defect is part of a two or three membered ring. This compares with a value of 102° found from bulk cluster calculations. Also, the vicinal Si-O-Si^II^ angle (where Si^II^ corresponds to the 2-fold coordinated silicon atom) is found to be between 90 and 133° in our nanoscale clusters against the corresponding values for the same defective site of 141–161° from bulk cluster calculations.

We note that for silica nanoclusters, relaxation is found to have little impact on the energetics of oxygen removal. An analysis of Figure 1 (see Table S1 for the values) shows that E_rel_ is generally between 0.06 and 0.12 eV, and is thus < 5% of the corresponding E_vac_. The structures of the relaxed O-deficient nanoclusters are very similar to the original stoichiometric structures. The only exception is the nanocluster structure for *N* = 12 that possesses two NBO sites. Oxygen vacancies originating from 2-fold coordinated oxygen atoms, which at nanoscale are less energetically favored than those generated from mono-coordinated oxygen sites, are also well-studied for bulk silica in the literature (Skuja et al., 1984; Skuja, 1998; Sulimov et al., 2002). Specifically, the ≡Si-Si≡ equilibrium distance is reported to be 2.3–2.5 Å in α-quartz (Skuja et al., 1984; Skuja, 1998; Sulimov et al., 2002). At the nanoscale, after removal of two-coordinated oxygens we find the ≡Si-Si≡ equilibrium distance to be between 2.12–2.37 Å, slightly lower with respect to the range reported for the bulk. Moreover, bulk oxygen removal is known to induce a long range structural distortion and asymmetric relaxation of atoms surrounding the vacancy (Sulimov et al., 2002). Such long-range effects are more difficult to evaluate for clusters for which the sizes range between 5 and 20 Å. However, generally we observe a relatively strong local relaxation for the neighboring atoms, whereas no significant long-range relaxation is observed (usually < 0.1 Å of total displacement for distances >3 Å from the vacancy).

The electronic structure of the oxygen-deficient non-stoichiometric silica clusters is consistently found to be a singlet, as expected. The electron density of the highest occupied state is mainly located on the O-deficient silicon site and has the shape of a *sp* orbital, see Figure S3.

### Pure (TiO_2_)_N_ Nanoclusters

**Figure 2:**
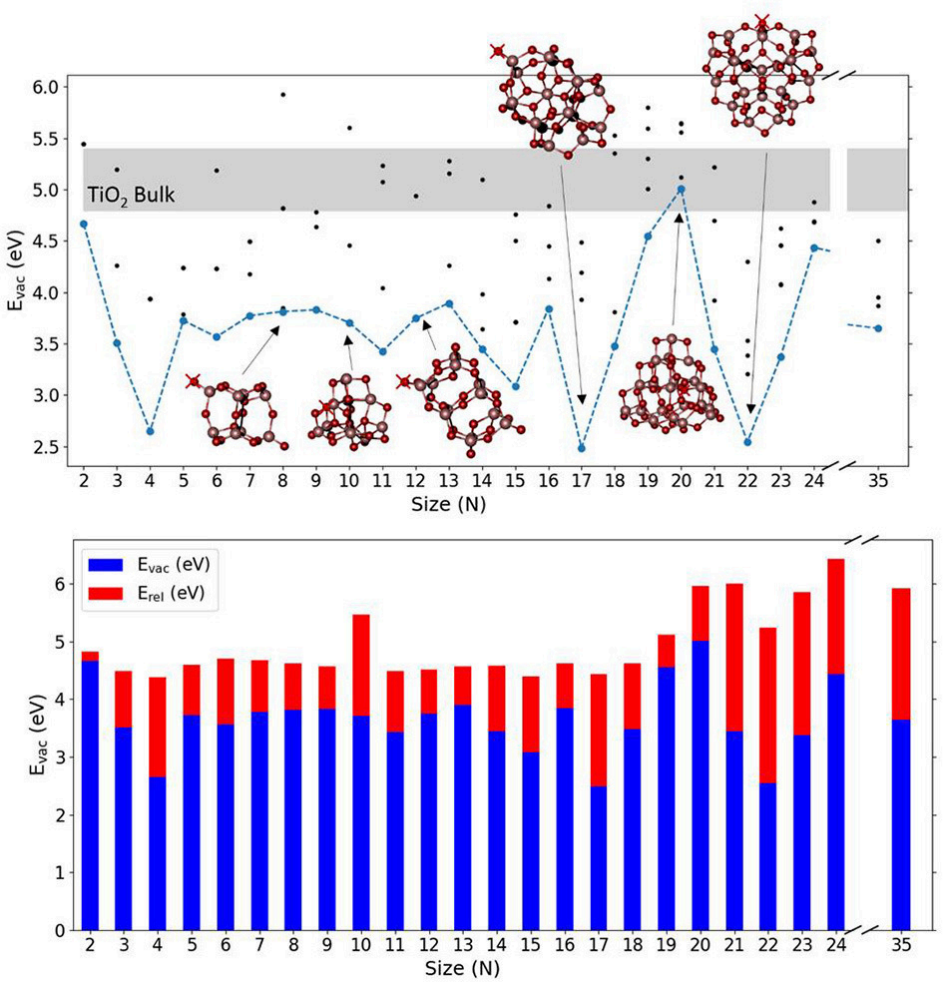
Top: E_vac_ as a function of the system size (N) for the considered (TiO_2_)_N_ nanoclusters. Blue line is relative to lowest E_vac_ obtained associated with the removal of O atoms. Black points indicate a selection of E_vac_ values obtained from the removal of different sites of O atoms. Light gray region indicates the range of calculated TiO_2_ bulk E_vac_ values reported in literature (see text). A selection of nanocluster structures is also shown where red crosses indicate the oxygen site that, upon removal, leads to the lowest E_vac_ value. Values of E_vac_ and E_rel_ for the nanoparticle with *N* = 35 are obtained from Kim et al. (2016). Bottom: the corresponding oxygen vacancy formation energies, E_vac_ (blue), and relaxation energies, E_rel_ (red), with respect to nanocluster size.

Figure 2 shows the calculated values of E_vac_ for the lowest energy (TiO_2_)_N_ nanoclusters together with selected nanocluster structures. Table S1 shows the corresponding values of E_vac_, E_unrel_, and E_rel_ obtained and Figure S4 shows the (TiO_2_)_N_ structures used. It can be observed that the (TiO_2_)_N_ nanocluster structures are more compact and less symmetric than silica clusters for the range selected, with titanium atoms being in 4-, 5-, and 6-fold coordinated environments. Many of these nanocluster structures, especially for sizes smaller than *N* = 20, possess terminal oxygen sites bonded to a Ti atom in a tetrahedral environment (≡Ti–O species). If present, these terminal oxygens are generally the least energetically costly to remove. In some cases (e.g., *N* = 4, 9, 11, 12), although the removal of a terminal oxygen does not have the lowest associated E_unrel_ value, removing a 2-fold oxygen atom nearby can lead to a reduced nanocluster with the same topology as the one that results from removing the terminal oxygen itself. E_vac_ values for (TiO_2_)_N_ nanoclusters are found to oscillate between 2.48 and 5.01 eV and display no clear size-dependent trend, contrary to the case of silica nanoclusters. The energetic cost to remove an oxygen atom seems to depend critically on the structure of each cluster. In very few cases (i.e., *N* = 17, 22), the E_vac_ values for TiO_2_ nanoclusters was found to be comparable with that found for SiO_2_ nanoclusters. In such cases, after the relaxation, the TiO_2_ nanocluster structure was found to have neither terminal oxygen sites (usually the site of the oxygen atom removal) nor 3-fold coordinated Ti defective centers, known to be highly destabilizing when left after the removal of a terminal site. We were not able to obtain a E_vac_ value for bulk systems with the computational setup used for the nanoclusters due to severe convergence problems associated with the presence of Ti^3+^ centers. Other authors have calculated E_vac_ for bulk and surfaces of anatase and rutile using DFT-based calculations employing hybrid functionals incorporating between 20 and 25% Hartree Fock exchange, obtaining values between 4.8 eV (Finazzi et al., 2008; Yamamoto and Ohno, 2012; Deák et al., 2014) and 5.4 eV (Islam et al., 2007; Janotti et al., 2010; Lee et al., 2012). We also note that the E_vac_ value for a terminal oxygen in an anatase bulk cut (TiO_2_)_35_ nanoparticle was calculated to be 3.95 eV, using the same methodology as employed herein (Kim et al., 2016). This value is similar to the average value of 3.65 eV, calculated for the lowest E_vac_ values over our set of nanoclusters. Interestingly, for the (TiO_2_)_35_ nanoparticle, the removal of an internal bulk-like three-coordinated oxygen resulted in a lower E_vac_ (i.e., 3.65 eV) with respect to that for removing a terminal oxygen atom. This behavior probably derives from the strong geometrical relaxation induced from the vacancy to the already metastable anatase bulk cut structure.

The relaxation of the TiO_2_ nanoclusters after oxygen removal is found to involve more energy than in the case of silica nanoclusters. Specifically, the average E_rel_ value for the lowest energy clusters is found to be 1.50 eV, and in some cases it accounts for more than 2.5 eV (*N* = 21, 23). With a few exceptions, such as for *N* = 4, 17, 22, the removal of terminal oxygens induces a moderate degree of local relaxation. However, when 2-fold coordinated oxygen atoms are removed, significant structural rearrangements take place (i.e., *N* = 10, 20, 21, 23, 24). In Figure S3 we show the O-deficient structure for *N* = 11 where a 2-fold oxygen atom is removed in the presence of a terminal oxygen, which then subsequently moves during the relaxation to fill the vacancy.

The electronic structure of the O-deficient clusters is either a triplet with two unpaired electrons occupying two titanium sites or an open shell singlet with unpaired electrons equally delocalized around three Ti centers neighboring the vacancy. Figure S3 displays the electron density associated with the highest occupied state of the (TiO_2_)_10_ and (TiO_2_)_11_ nanocluster after the removal of an oxygen atom, which is localized on Ti^3+^ sites that carry the electrons left in the vacancy.

### Mixed (Si_x_Ti_1–x_O_2_)_*N*_ Nanoclusters

**Figure 3:**
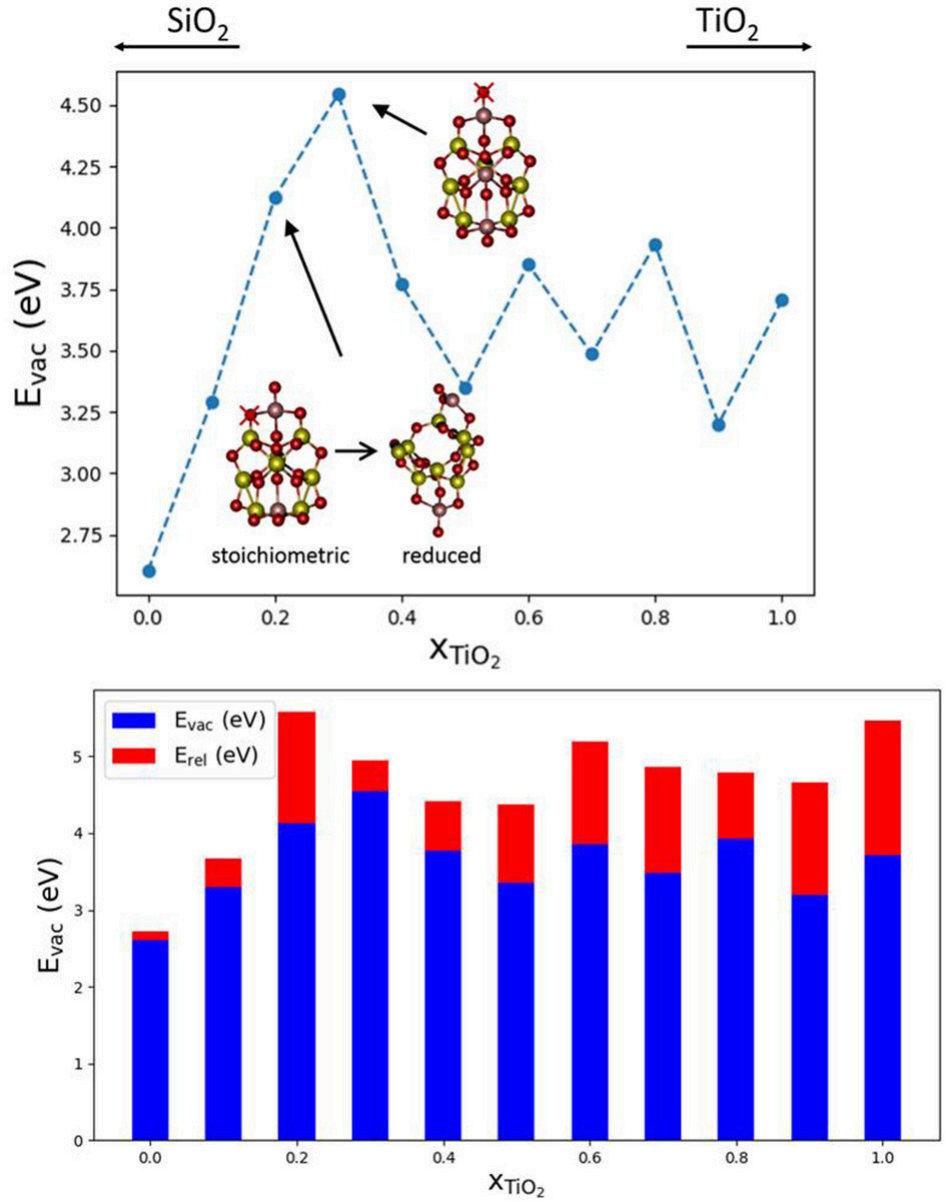
Top: E_vac_ vs. x plot; global minima isomers for (Ti_x_Si_1−x_O_2_)_10_ with 0 < *x* < 1, the red crosses indicate the O atom that, after removal, leads to the lowest E_vac_. Bottom, the corresponding oxygen vacancy formation energies, E_vac_ (blue), and relaxation energies, E_rel_ (red), with respect to nanocluster composition.

In order to study the effect of mixing SiO_2_ and TiO_2_ on E_vac_ values, we have selected (Ti_x_Si_1−x_O_2_)_10_ nanoclusters and a Ti content *x* between 0 and 1. The nanocluster structures used are taken from the global optimized structures reported in Cuko et al. (2018) (see also Figure S5). The nanoclusters exhibit different isomers for pure and mixed structures. All the structures possess terminal ≡Ti-O oxygen sites, with the exception of the pure (TiO_2_)_10_ nanocluster. The pure silica (SiO_2_)_10_ nanocluster displays only silanone terminations, whereas mixed systems display typically two terminal oxygen centers bonded with titanium atoms. The presence of titanium thus seems to allow for relatively more energetically stable terminal oxygen sites than silicon (Cuko et al., 2018). Figure 3 shows that the E_vac_ values obtained for the mixed nanocluster systems oscillate around 3.7 eV, with the lowest value being found for *x* = 0.1 (E_vac_ = 3.29 eV) and the highest value for *x* = 0.3 (E_vac_ = 4.54 eV). The mixed nanocluster structure with *x* = 0.1 possesses two terminal oxygen sites bound to a Si and Ti atom. The removal of the terminal oxygen is more energetically facile (i.e., E_vac_ is relatively lower) when it is bound to a silicon atom rather than a titanium atom. As the content of TiO_2_ increases, the E_vac_ values are found to initially increase up to *x* = 0.30, and afterwards decrease and oscillate with no obvious trend.

Over the range of (Ti_x_Si_1−x_O_2_)_10_ nanoclusters, relaxation effects are relatively less significant for clusters with titania contents of *x* = 0.0, 0.1, 0.3. In the first two cases, indeed, the less energetically costly oxygen to be removed is from a silanone or a Si-NBO site, and therefore, the local structural relaxation effects are similar to those of pure silica systems. The system with *x* = 0.2 is a special case since lowest E_vac_ value we could find is related to the removal of a two coordinated oxygen as shown. The removal of this oxygen atom leads to a significant structural change during the geometry optimization with a consequent relatively large associated E_rel_ value (see Figure 3). For higher Ti content, E_rel_ energies are found to be in the range of 0.6–1.5 eV which is typical for pure TiO_2_ nanoclusters. Overall, the mixed (Ti_x_Si_1−x_O_2_)_10_ nanocluster systems tend to behave more like titania nanoclusters rather than silica nanoclusters in terms of reduction through O-vacancy formation.

## Discussion

The results obtained point to a (sub)nanosize dependency of the formation of oxygen vacancies in silica- and titania-based structures. Firstly, the removal of singly-coordinated terminal oxygen atoms is often associated with low E_vac_ values. This is explained by the low energetic cost of breaking an M-O bond compared with the energy required to break two M–O bonds for oxygen atoms in 2-fold coordinated sites. This is observed in the three types of nanoclusters studied in this work and has been reported in the literature for other oxides, especially in systems exhibiting structural stable dangling bonds. For instance, V_2_O_5_ in bulk, surface and clusters exhibit structurally stable terminal oxygen sites. The coordination of oxygen sites in vanadia is commonly associated with chemical reactivity (Calatayud and Minot, 2006; Ganduglia-Pirovano et al., 2007; Calatayud et al., 2008b). The lower E_vac_ found for nanoclusters compared to bulk values indicates higher reducibility of the nanosized materials as has been reported for other oxide systems like CeO_2_ and ZrO_2_ (Bruix and Neyman, 2016; Ruiz Puigdollers et al., 2016b).

**Figure 4:**
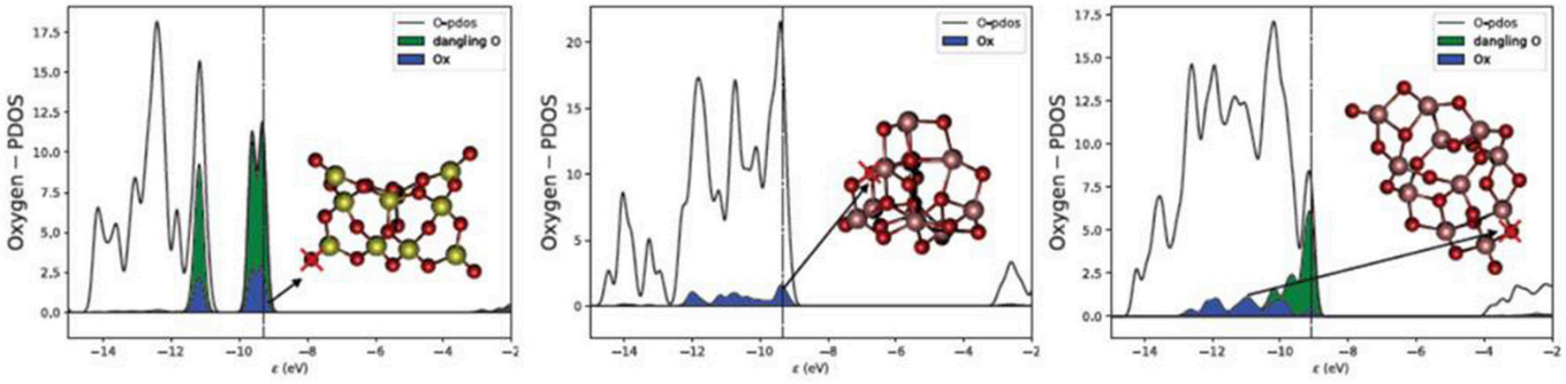
Projected density of states (PDOS) on for oxygen atoms for (SiO_2_)_10_ (left), (TiO_2_)_10_ (middle), and (TiO_2_)_11_ (right). Ox indicates the projection on the oxygen center marked with a cross (blue). Dangling O indicates the projection on all single terminal oxygen sites (green). Vertical lines indicate the position of the Fermi level. The PDOS plot has been smeared for clarity.

It is, however, worth noting that the lowest E_vac_ values found for silica clusters, 2.3–3.6 eV, are generally lower than for titania clusters, 2.48–5.01 eV as also shown from Figures 1, 2 and Table S1. A direct comparison between bulk SiO_2_ and bulk TiO_2_ was not possible in this study due to the high computational cost and convergence problems with TiO_2_ systems. However, there are several studies on O vacancy defects formation in metal oxides (Pacchioni, 2008; Deml et al., 2015; Helali et al., 2017) that agree on associating a higher reducibility to titania (either rutile and anatase phases) with respect to silica bulk (mainly α-quartz). Our results suggest that silica becomes more reducible than titania at the nanoscale, which is contrary to the properties observed for respective bulk materials. The reason for such inverted behavior seems to be connected to the presence of terminal silanone oxygen atoms in low energy silica nanoclusters. The electronic structure (projected density of states) of this site is displayed in Figure 4, showing that the energy levels are highly localized in two sharp peaks (one between −9 and −10 eV, and the other between −10.5 and −11.5 eV) associated to higher reactivity. In comparison, the oxygen levels of the 2-fold oxygen in (TiO_2_)_10_ are distributed over a 3 eV range of energies (between −9 and −12 eV). In the (TiO_2_)_11_ cluster, the electronic energy levels associated with the dangling oxygen lay closer to the Fermi level, between −9 and −11 eV, and those associated with the 2-fold coordinated site levels spread between −9 and −13 eV. The electronic structure of the dangling silanone oxygen in silica is consistent with an enhanced reactivity of this site and with the low E_vac_ values shown. Interestingly, the oxygen vacancy in bulk silica has been reported to exhibit specific electronic transitions associated with the electron pair localized on the silicon atom after reduction (Cannizzo and Leone, 2004; Sushko et al., 2005). We also observe the highest occupied electronic level of O-deficient (SiO_2_)_10_ cluster localized on the resulting Si < site (Figure S3 top right), which is consistent with the presence of an electron pair localized on the silicon site.

A second important point is the structure-dependency of the E_vac_ values. In the case of silica nanoclusters, the E_vac_ value is almost constant and related to the presence of Si sites with dangling silanone oxygen sites. However, titania nanoclusters display a greater range of E_vac_ values, depending on the structure near to the removed oxygen atom. Relaxation effects can also be significantly stronger in titania nanoclusters than in silica nanoclusters, which is consistent with the greater variety of E_vac_ sites and values found. The size dependency of E_vac_ values is also different for each nanomaterial. For the system sizes considered here, the majority of silica nanoclusters exhibit a near constant value of E_vac_ ~ 2.6 eV associated with silanone sites, and exceptionally an E_vac_ value of 3.6 eV associated with the NBO in the (SiO_2_)_12_ nanocluster. For larger sizes, silica nanoclusters are predicted to become fully-coordinated (Flikkema and Bromley, 2009), which will result in a jump of E_vac_ to near bulk values ~5 eV. As can be seen in Figure S2, 2-fold coordinated oxygens cost 4.4–5.5 eV. In the case of pure titania nanoclusters there seems to be no clear trend with size.

Finally, mixing nanosilica and nanotitania results in chemically and structurally rich nanocluster systems displaying a variety of oxygen sites. Interestingly the presence of titanium induces the disappearance of silanone sites in favor of pyramidal ≡Ti–O species. Whereas terminal oxygen sites are relatively unstable when bonded to silicon, they are stabilized when bonded to titanium. As for pure titania nanoclusters, the values of E_vac_ are found to strongly depend on the structure, the coordination of the oxygen removed, and the degree of relaxation after oxygen removal.

**Figure 5:**
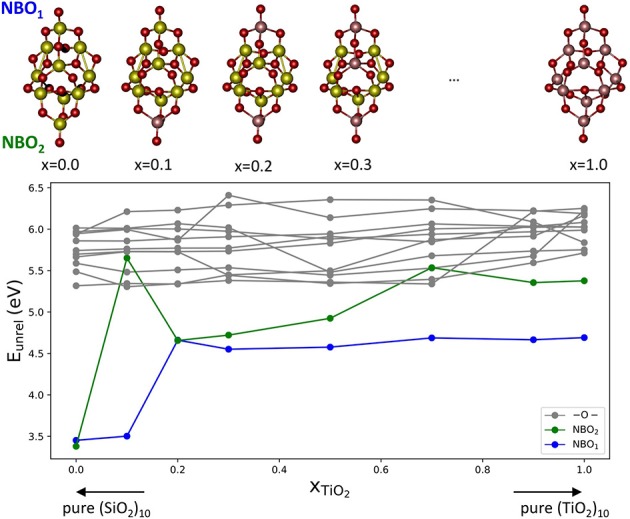
Top: a set of (Ti_x_Si_1−x_O_2_)_10_ cluster structures exhibiting the same structural isomer for pure silica (*x* = 0), pure titania (*x* = 1) and all intermediate compositions. Bottom: the corresponding oxygen formation energy without relaxation (E_unrel_) in eV for all non-equivalent oxygen sites. Vacancies are generated after removing a 2-fold coordinated oxygen (gray), terminal NBO_1_ (blue), and terminal NBO_2_ (green).

In order to further study the role of the chemical nature of Si and Ti in the oxygen vacancy energy, we consider a set of (Ti_x_Si_1−x_O_2_)_10_ structural isomers with the same topology (Figure 5), with selected compositions between *x* = 0–1. Such isomer structures are global minima for titanosilicates with *x* = 0.1, 0.2, and 0.3, and are slightly less stable than the global minima for pure systems. They have been geometrically optimized in their stoichiometric composition. We then remove each non-equivalent oxygen atom keeping the initial geometry frozen. In Figure 5 we report the resulting unrelaxed oxygen vacancy formation energies (E_unrel_).

Figure 5 shows E_unrel_ as a function of the mixing composition x_TiO2_ of the cluster. In the plot there are three families of oxygen vacancies according of the type of oxygen removed: the vacancies originating from the removal of two-coordinated oxygen atoms are denoted in gray, whereas those originating from removing the terminal oxygen sites labeled NBO_1_ and NBO_2_, respectively, are denoted in blue and green. In most of the cases, vacancies at terminal position are found to provide the lowest E_unrel_ value. For the pure silica cluster isomer, the E_unrel_ values for the NBO_1_ and NBO_2_ are 3.38 and 3.45 eV, respectively, which values are significantly lower than the corresponding values for pure titania systems, 4.69 and 5.38 eV. This result further confirms that the oxygen vacancy formation energy is driven by the presence of unsaturated O centers and, particularly when such centers are bonded to Si atoms, are more easily removed than when they are bonded to Ti atoms. This is clearly seen in the *x* = 0.1 isomer that possesses one terminal oxygen on Si and one on Ti: the E_unrel_ for the Si-bonded oxygen NBO_1_ is 3.50 eV whereas for the Ti-bonded oxygen NBO_2_ it is 5.65 eV. Although relaxation effects have not been considered, based on the results discussed above, they are not expected to be significant. Therefore, the nature of the cationic site, Si or Ti, seems to critically determine the stabilization of the terminal oxygen deficient systems.

## Conclusions

In this work we use DFT-based calculations employing the PBE0 hydrid functional to explore oxygen vacancy formation in globally optimized TiO_2_, SiO_2_, and mixed Ti_x_Si_1−x_O_2_ nanoclusters for sizes between 2 and 24 stoichiometric oxide units. The properties computed (oxygen vacancy formation energies, electronic structure, nanocluster structure, relaxation effects) are found to critically depend on the nature of the oxide. The behavior of silica nanoclusters is found to be rather constant and dominated by the presence of dangling oxygens in silanone >Si = O sites. In titania and titanosilicate nanoclusters; however, we find a strong dependence of the properties with respect to the local geometry. When unsaturated oxygen centers are present in the system, removing such centers is energetically less costly than removing two-coordinated oxygen atoms. Since at the nanoscale such unsaturated centers are naturally present in low energy systems, contrary to the bulk and extended surfaces, we can predict that the reduction of nanosized silica clusters is energetically more favorable than in the bulk. Furthermore, we found that the oxygen vacancy formation in silica nanoclusters is also more favorable than from similar-sized low energy titania nanoclusters. This demonstrates the emergence of an unexpected chemical behavior induced by the small size of the systems considered. We hope that our results will help in the search of novel materials for applications in scientific and technological fields such as heterogeneous catalysis, electronics, and cluster chemistry.

## Author Contributions

AC performed all the calculations, figures, tables, analyzed the data, discussed results, and participated in the elaboration of the manuscript. SB and MC directed the work, discussed the data, and wrote the manuscript.

### Conflict of Interest Statement

The authors declare that the research was conducted in the absence of any commercial or financial relationships that could be construed as a potential conflict of interest.

## Supplementary Material

Supplementary Data Sheet 1 Oxygen vacancy formation energies with and without relaxation (E_vac_, E_unrel_, E_rel_), nanocluster structures employed, E_vac_ values for different O-vacancies, electron density plots of selected O-deficient structures.
