# Supplementary material for: Oxygen Vacancies in Oxide Nanoclusters: When Silica Is More Reducible Than Titania
Source: Front Chem. 2019 Feb 7;7:37. doi: 10.3389/fchem.2019.00037 (PMC6374336; doi:10.3389/fchem.2019.00037)

Supplementary material :

Oxygen vacancies in oxide nanoclusters: when silica is more reducible than titania

Andi Cuko[1,2], Stefan T. Bromley[1,3], Monica Calatayud[2,*]

[1] Departament de Ciència de Materials i Química Física Institut de Química Teòrica i Computacional (IQTCUB), Universitat de Barcelona, E-08028 Barcelona, Spain

[2] Sorbonne Université, CNRS, Laboratoire de Chimie Théorique, LCT, F. 75005 Paris, France

[3] Institució Catalana de Recerca i Estudis Avançats (ICREA), E-08010 Barcelona, Spain

Figure S1 : Structures of silica nanoclusters employed.

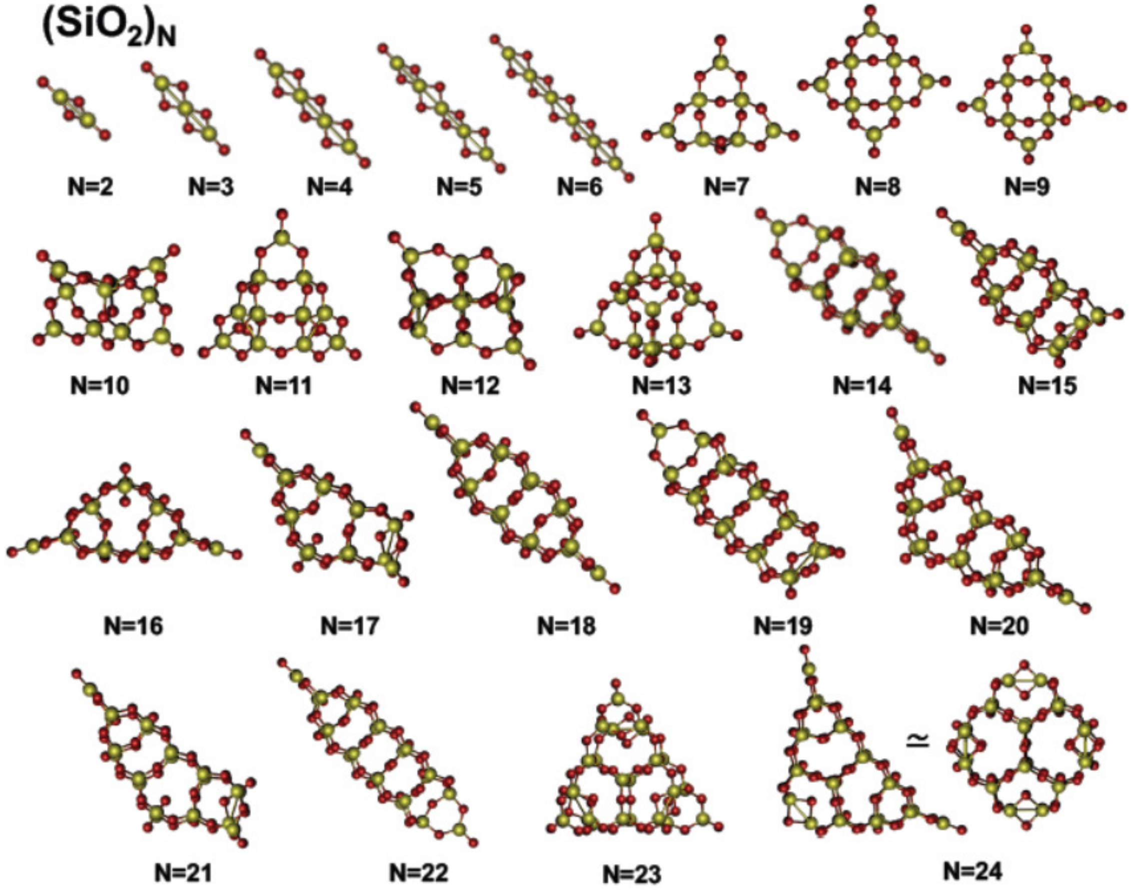

Table S1 : Calculated values of E_{unrel}, E_{vac} and E_{rel} for the most stable reduced clusters found.

**N $(SiO_2)_N$**	E_{unrel}	E_{vac}	E_{rel}
2	2.43	2.37	0.06
3	2.50	2.42	0.08
4	2.52	2.44	0.08
5	2.53	2.46	0.07
6	2.54	2.46	0.08
7	2.69	2.57	0.12
8	2.72	2.60	0.12
9	2.56	2.47	0.09
10	2.72	2.60	0.12
11	2.66	2.53	0.13
12	3.60	3.36	0.24
13	2.70	2.58	0.12
14	2.77	2.65	0.12
15	2.73	2.61	0.12
16	2.77	2.65	0.12
17	2.74	2.62	0.12
18	2.76	2.64	0.12
19	2.73	2.62	0.11
20	2.76	2.65	0.11
21	2.73	2.61	0.12
22	2.75	2.64	0.11
23	2.82	2.70	0.12
24	2.98	2.86	0.12
**N $(TiO_2)_N$**	E_{unrel}	E_{vac}	E_{rel}
2	4.83	4.67	0.16
3	4.49	3.50	0.99
4	4.39	2.65	1.74
5	4.59	3.72	0.87
6	4.70	3.57	1.13
7	4.68	3.77	0.91
8	4.63	3.81	0.82
9	4.56	3.83	0.73
10	5.47	3.71	1.76
11	4.49	3.42	1.07
12	4.51	3.75	0.77
13	4.57	3.90	0.67
14	4.58	3.44	1.14
15	4.39	3.08	1.31
16	4.62	3.84	0.78
17	4.44	2.48	1.96
18	4.62	3.48	1.14

19	5.12	4.55	0.57
20	5.96	5.01	0.95
21	6.00	3.44	2.56
22	5.24	2.54	2.70
23	5.86	3.37	2.49
24	6.44	4.44	2.00
**x $(Ti_xSi_{1-x}O_2)_{10}$**	**E_{unrel}**	**E_{vac}**	**E_{rel}**
0.0	2.72	2.60	0.12
0.1	3.66	3.29	0.37
0.2	5.58	4.12	1.46
0.3	4.95	4.54	0.41
0.4	4.42	3.77	0.65
0.5	4.37	3.34	1.02
0.6	5.19	3.85	1.34
0.7	4.86	3.49	1.37
0.8	4.79	3.93	0.85
0.9	4.66	3.20	1.46
1.0	5.47	3.71	1.76

Figure S2 : Selected nanocluster structures for $(SiO_2)_{10}$ (left), $(TiO_2)_{10}$ (middle), and $(TiO_2)_{11}$ (right). Arrows indicate the oxygen atom to be removed and the values correspond to the corresponding E_{vac} value in eV.

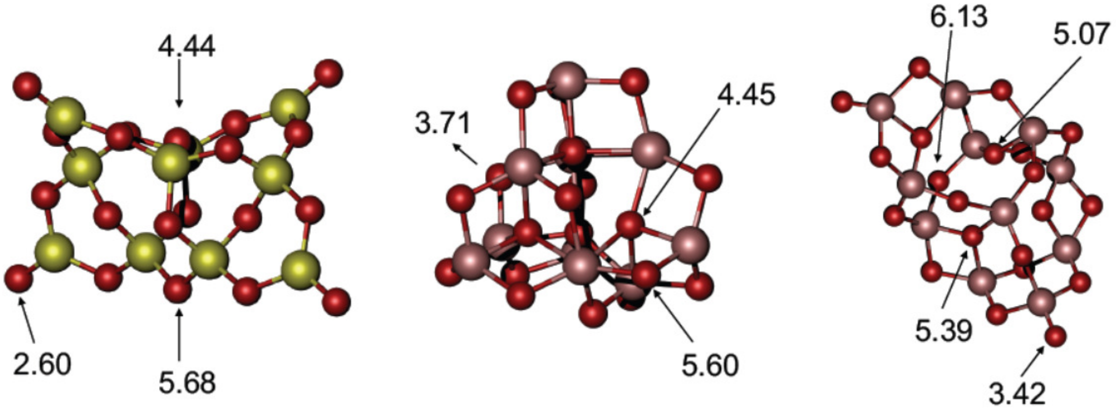

Figure S3 : Schematic reduction reaction for $(SiO_2)_{10}$ (top), $(TiO_2)_{10}$ (middle), and $(TiO_2)_{11}$ (bottom). The electron density of the highest occupied state of the reduced cluster is also shown on the right of each reaction.

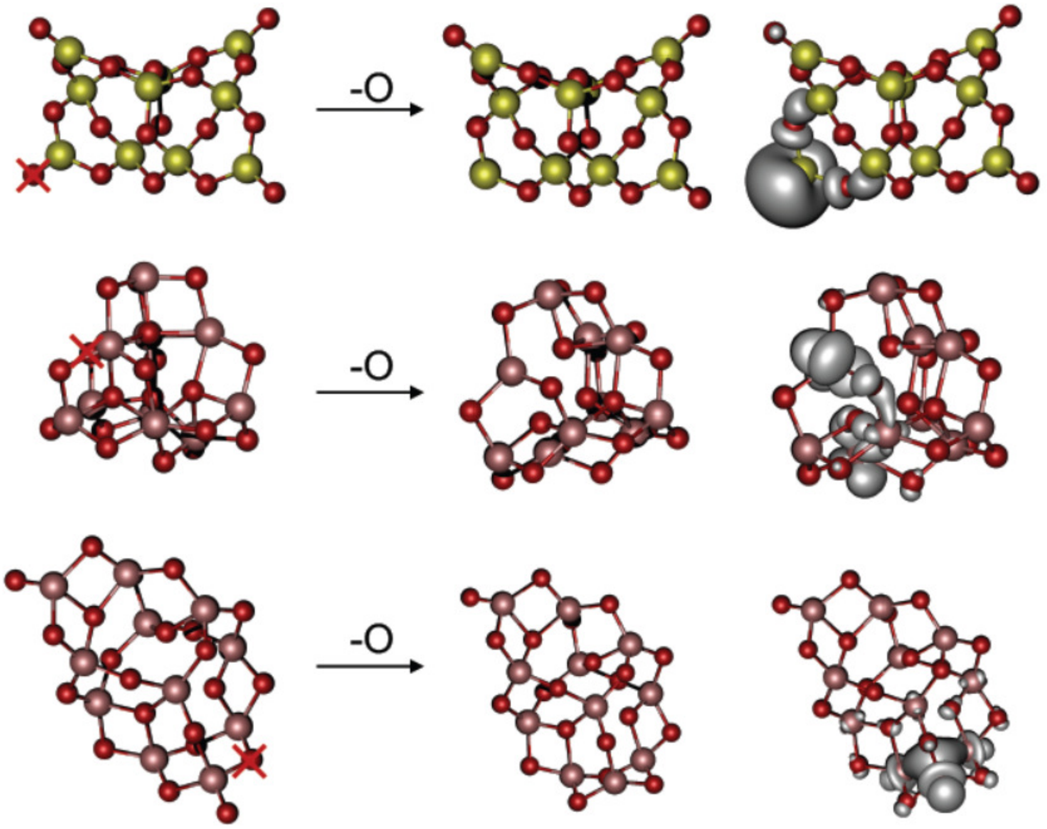

Figure S4 : Structures of titania nanoclusters employed.

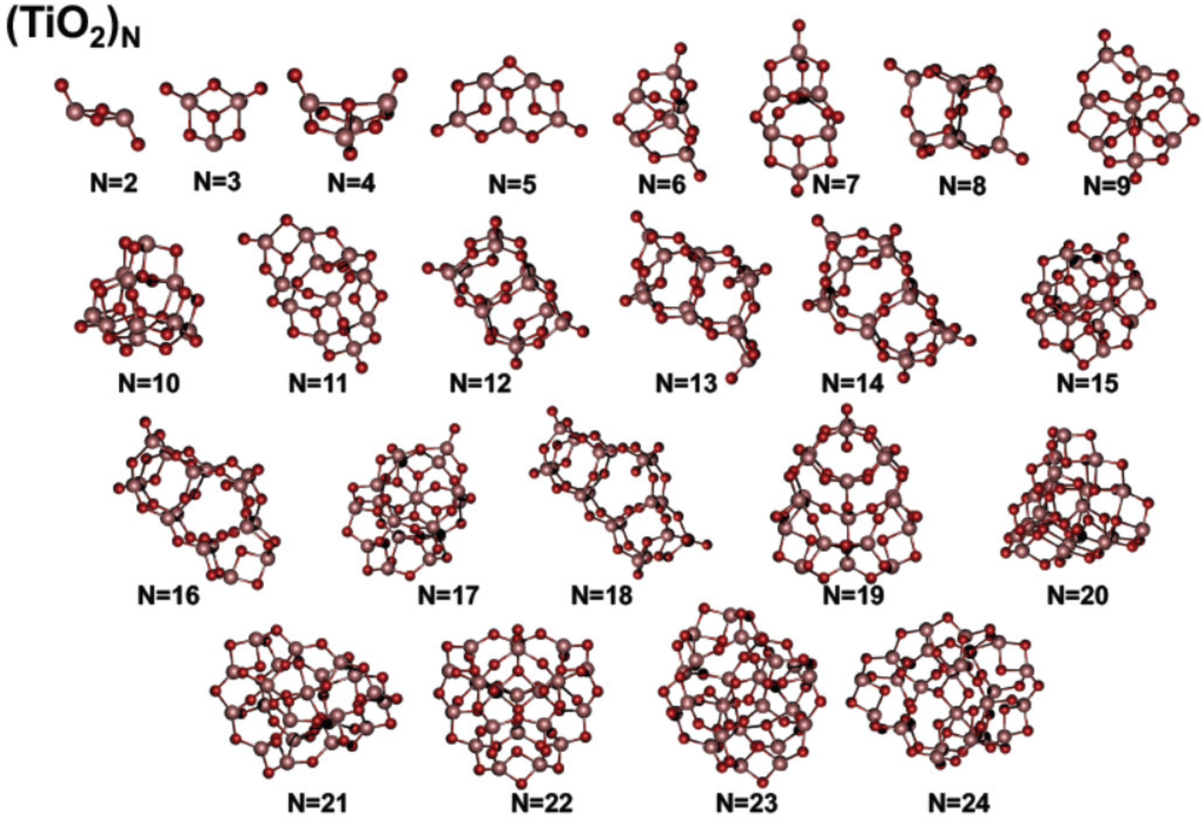

Figure S5 : Structures of mixed $(Ti_xSi_{1-x}O_2)_{10}$ nanoclusters employed.

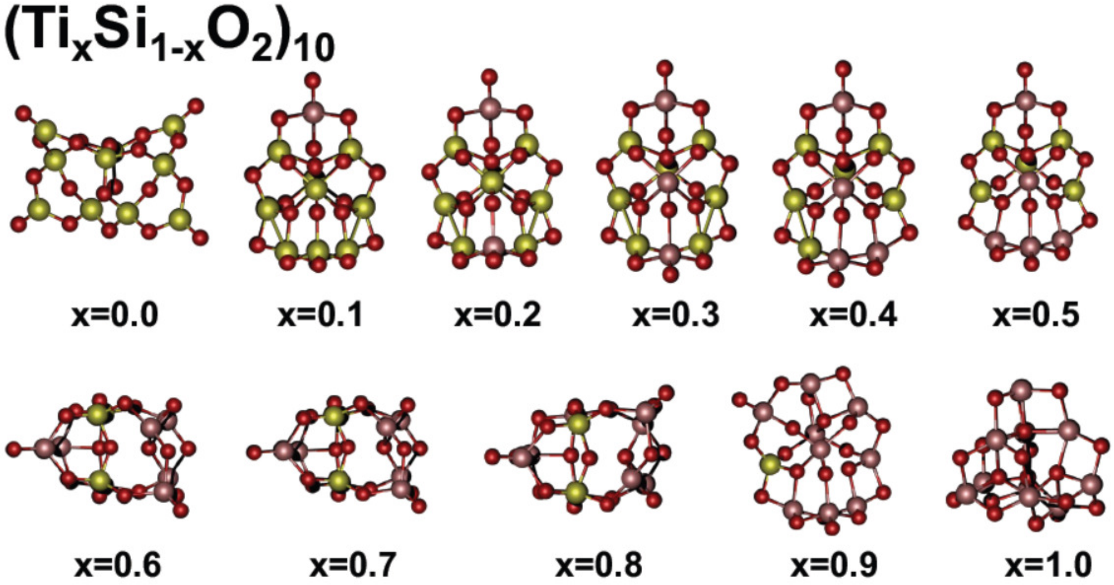

Supplement: Supplementary Data Sheet 1 — Oxygen vacancy formation energies with and without relaxation (Evac, Eunrel, Erel), nanocluster structures employed, Evac values for different O-vacancies, electron density plots of selected O-deficient structures. [file Data_Sheet_1.PDF]
